# Mitochondrial activity in gametes and transmission of viable mtDNA

**DOI:** 10.1186/s13062-015-0057-6

**Published:** 2015-05-16

**Authors:** Liliana Milani, Fabrizio Ghiselli

**Affiliations:** Dipartimento di Scienze Biologiche, Geologiche ed Ambientali, Università di Bologna, Via Selmi 3, 40126 Bologna, Italy

**Keywords:** Mitochondrial membrane potential, Mitochondrial inheritance, Doubly uniparental inheritance of mitochondria, Germ line mitochondria, Oxidative phosphorylation, Reactive oxygen species, Oxidative stress, Ageing, mtDNA selection, Balbiani body

## Abstract

**Background:**

The retention of a genome in mitochondria (mtDNA) has several consequences, among which the problem of ensuring a faithful transmission of its genetic information through generations despite the accumulation of oxidative damage by reactive oxygen species (ROS) predicted by the free radical theory of ageing. A division of labour between male and female germ line mitochondria was proposed: since mtDNA is maternally inherited, female gametes would prevent damages by repressing oxidative phosphorylation, thus being quiescent genetic templates. We assessed mitochondrial activity in gametes of an unusual biological system (doubly uniparental inheritance of mitochondria, DUI), in which also sperm mtDNA is transmitted to the progeny, thus having to overcome the problem of maintaining genetic information viability while producing ATP for swimming.

**Results:**

Ultrastructural analysis shows no difference in the conformation of mitochondrial cristae in male and female mature gametes, while mitochondria in immature oocytes exhibit a simpler internal structure. Our data on transcriptional activity in germ line mitochondria show variability between sexes and different developmental stages, but we do not find evidence for transcriptional quiescence of mitochondria. Our observations on mitochondrial membrane potential are consistent with mitochondria being active in both male and female gametes.

**Conclusions:**

Our findings and the literature we discussed may be consistent with the hypothesis that template mitochondria are not functionally silenced, on the contrary their activity might be fundamental for the inheritance mechanism. We think that during gametogenesis, fertilization and embryo development, mitochondria undergo selection for different traits (e.g. replication, membrane potential), increasing the probability of the transmission of functional organelles. In these phases of life cycle, the great reduction in mtDNA copy number per organelle/cell and the stochastic segregation of mtDNA variants would greatly improve the efficiency of selection. When a higher mtDNA copy number per organelle/cell is present, selection on mtDNA deleterious mutants is less effective, due to the buffering effect of wild-type variants. In our opinion, a combination of drift and selection on germ line mtDNA population, might be responsible for the maintenance of viable mitochondrial genetic information through generations, and a mitochondrial activity would be necessary for the selective process.

**Reviewers:**

This article was reviewed by Nick Lane, Fedor S Severin and Fyodor Kondrashov.

## Background

Following the endosymbiotic event that originated mitochondria, the vast majority of genes from the protomitochondrion have been transferred to the nucleus or lost [1]. Metazoan mitochondrial DNA (mtDNA) is very compact and typically consists of 13 genes encoding proteins involved in oxidative phosphorylation (OXPHOS), 24 genes involved in mtDNA translation (2 rRNAs and 22 tRNAs), and a control region for mtDNA replication and transcription, so, although mitochondria are involved in multiple cellular processes, the information stored in their genome is limited, and concerns only OXPHOS activity (see [2] for exceptions). Why did mitochondria retain a genome? Why is its content extremely conserved among Metazoa? Why only OXPHOS genes? According to the “co-location for redox regulation” hypothesis (CoRR), the retention of OXPHOS genes provides mitochondria with a local and autonomous control of their expression by the redox state of electron transport chain complexes [3,4]. The result is an increased and fine-tuned energy production that releases the eukaryotic cell from genome size constraints, making mitochondria a prerequisite to eukaryote complexity and multicellular life [5,6].

The retention of a genome in the mitochondrion entails three major issues: 1) mitochondrial and nuclear products (proteins and RNAs) interact to carry out their function, so the two genomes need to coevolve despite their markedly different evolutionary dynamics (i.e. substitution rate, recombination and population size; see [7-9] for review); 2) the potential evolution of selfish mitochondrial variants leading to genomic conflicts [10]; 3) OXPHOS is accompanied by the generation of mutagenic reactive oxygen species (ROS), so mtDNA is located in a potentially hostile environment, which can compromise the integrity of the genetic information [11]. The free radical theory of ageing [12] posits that somatic mutations occurring in mtDNA, by affecting the efficiency of respiratory proteins, result in an increase of ROS production, which in turn increases the mutation rate (see [13] for a review). However, it was observed that the physiological level of ROS in normally functioning mitochondria is negligible thanks to defense systems that regulate ROS generation and removal (see [14], for a review).

The evolution of uniparental inheritance helps to optimize mitonuclear match by promoting homoplasmy, because all mitochondria derive from one gamete only, through a “mitochondrial bottleneck” that reduces mtDNA variability (discussed in detail in [15]). Moreover, uniparental inheritance prevents genomic conflicts and the spread of cytoplasmic selfish elements [10,16,17]. Although uniparentality represents a viable solution for the first two issues, it is not sufficient to explain how the genetic information stored in mtDNA can be faithfully transmitted through generations, despite the accumulation of oxidative damage predicted by the free radical theory of ageing (point 3 above). Mitochondria cannot be produced *de novo* by the cell, but are inherited through generations, so high mutation rate, oxidative damage and lack of sexual reproduction would lead to the fast accumulation of deleterious mutation, a phenomenon known as Müller’s ratchet. How are germ line mitochondria preserved from the degeneration of genetic information? [18] proposed a division of labour between male and female germ line mitochondria: on the one hand spermatozoa maximize their motility by sacrificing mtDNA to OXPHOS, because usually they do not transmit their mtDNA; on the other hand non-motile female gametes prevent damages by repressing OXPHOS, thus being quiescent genetic templates. Recently, studies on three phylogenetically distant species (*Aurelia aurita*, *Danio rerio* and *Drosophila melanogaster*) reported results consistent with this theory [19-21].

So far, the Doubly Uniparental Inheritance of mitochondria (DUI), observed in some bivalve molluscs, is the only known evolutionary stable exception to Strictly Maternal Inheritance (SMI) typical of metazoans. In DUI animals, two mitochondrial lineages are inherited, one through eggs (F-type), the other through sperm (M-type) (reviewed in [22]): in male embryos, mitochondria derived from the spermatozoon form an aggregate that enters the primordial germ cells (PGCs), while in female embryos, sperm mitochondria are dispersed and/or degraded [23-26]. The two mitochondrial lineages have evolved independently for million years (for example >200 in unionids; see [27]), accumulating up to 50% of sequence divergence [22]. DUI females are generally homoplasmic for F, while males are heteroplasmic in their soma and homoplasmic for M in sperm. Since eggs do not transmit M [28,29], germ line mitochondria of DUI males are apportioned from the four/five mitochondria of the fertilizing spermatozoon [26], which carry a few hundred mitochondrial genomes [29] that must be functional and successfully inherited (proved by the long evolutionary persistence of DUI [22]). Thanks to its features, DUI provides a unique and evolutionary stable system to study mitochondrial inheritance, heteroplasmy, mitonuclear coevolution, genomic conflicts, and many basic aspects of mitochondrial biology [2,22,30]. Since in DUI species sperm mitochondria are inherited through male lineages, the need for M mitochondria to function both as energy supply for sperm swimming and as genetic templates represents an ideal situation to study the relationship between mitochondrial activity, mtDNA damage and faithful transmission of mitochondrial genetic information.

In the present work, we investigate the activity of germ line mitochondria in the DUI species *Ruditapes philippinarum* (the Manila clam), and we show that mitochondria are active in both male and female germ lines. We review and discuss our results in the light of the existing literature, and we propose that our findings are not specific of *R. philippinarum*: accordingly, mitochondrial activity in the germ line would not be harmful, but necessary for a correct germ line development, and also for the selective process that is responsible for the transmission of viable mtDNA through the generations.

## Results and discussion

How the viability of mitochondrial genome is preserved from generation to generation is an important and debated question. A better knowledge of the mechanisms that stand beyond a correct transmission of mitochondrial genetic information would greatly help the understanding of basic processes of cellular life, such as ageing, cell proliferation, cell differentiation, and gamete competence. Unfortunately, after decades of studies, the role of oxidative stress in the above-mentioned processes is still poorly understood and experimental evidence is controversial, because of the biological complexity of the subject and the technical difficulties in testing it.

Prompted by some recently published papers [19-21], we wanted to assess mitochondrial activity in male and female gametes of an unusual biological system, in which sperm mitochondrial DNA is inherited from father to son, thus having to maintain genetic information viability.

Female and male mature gonads of *R. philippinarum* (and of bivalves in general) consist of thousands of gonadic units called acini (for acinus morphology see [25]), a sort of sacks made of germinative epithelium supported by connective tissue [31]. Gametes in different stages of maturation within a single acinus can be seen, positioned in a centripetal way, with more peripheral immature stages and central mature gametes. In female acini, immature oocytes are localized at the periphery, connected to the acinus wall. During their maturation, oocytes become pedunculated and, approaching the time of spawning, they detach from the wall and fill the acinus lumen [25]. Spermatogenesis occurs centripetally with mature spermatozoa free and mobile within the acinus lumen [25].

### Mitochondrial ultrastructure in *R. philippinarum* gametes

**Figure 1:**
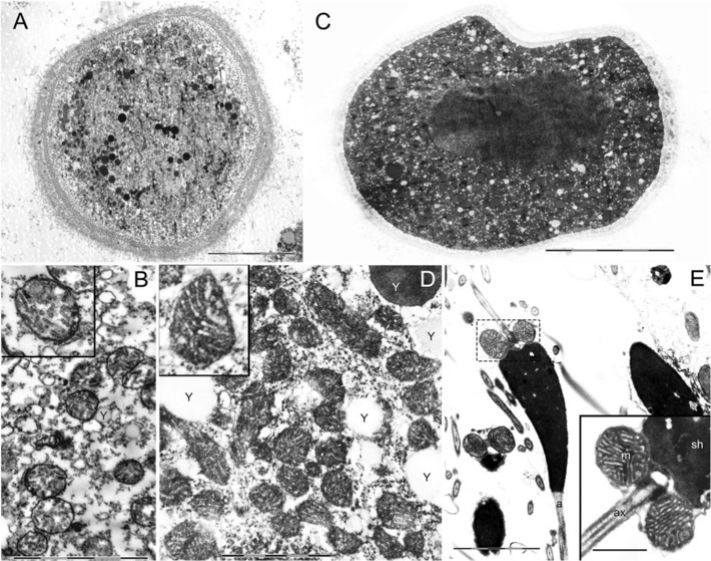
Mitochondrial ultrastructure at the transmission electron microscope (TEM). **(A)** Immature oocyte (scale bar = 10 μm). **(B)** Immature oocyte mitochondria. Oocytes just starting vitellogenesis show a simple internal structure with few and short mitochondrial cristae (scale bar = 2 μm). Yolk (Y). **(C)** Mature oocyte (scale bar = 20 μm). **(D)** Mature oocyte mitochondria. Vitellogenic oocytes show well formed mitochondrial cristae in mitochondria usually measuring less than 600 nm (scale bar = 1 μm). Yolk (Y). **(E)** Spermatozoa show well formed cristae in mitochondria measuring generally more than 700 nm (scale bar = 2 μm; inset = 0.7 μm). Sperm midpiece containing mitochondria (dashed box); acrosome (a). Inset: spermhead (sh); mitochondria (m); axoneme portion (ax).

Transmission Electron Microscope (TEM) analysis on immature oocytes (Figure 1A) showed mitochondria with simpler internal structure (Figure 1B), compared to mitochondria in male and female mature gametes. In fact, both vitellogenic oocytes (Figure 1C,D) and spermatozoon mitochondria (Figure 1E) had well developed cristae. No ultrastructural difference between male and female mature gametes in the conformation of mitochondrial cristae was visible, and organelle dimensions were in the usual range for this species [26]. This finding is consistent with previous TEM analyses on other animals in which mitochondria from fully grown oocytes typically show a dense matrix traversed by numerous cristae (see for example *Actinia fragacea* [32]). As documented by the large amount of TEM analyses on gametes, the mitochondrial ultrastructure during oocyte maturation changes considerably and also the experimental treatment deeply influences the resulting shape of these organelles (known at least since [33]). Using isotonic conditions, we are confident that the analyzed samples should have maintained the original morphology. That said, different stages of gamete development appear to have different internal structure throughout maturation.

It has to be mentioned that the mitochondrial ultrastructure *per se* does not appear to well represent organelle activity. In humans, for example, oocyte and early embryonic mitochondria look structurally undeveloped, showing few truncated cristae [34], but despite their seemingly primitive state, they perform OXPHOS and are the primary source of ATP in the oocyte and early embryo [35,36].

### Mitochondrial transcription in *R. philippinarum* gametes

**Figure 2:**
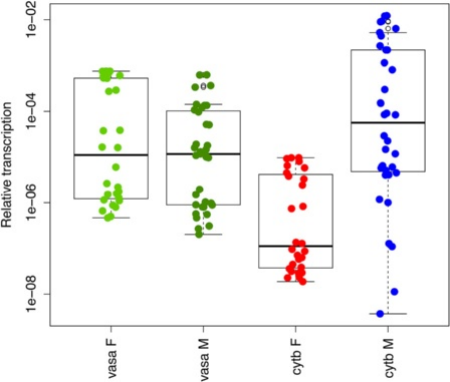
Transcript quantification of *vasa* and *cytb* genes in developing gonads obtained by RealTime qPCR. The *vasa* gene shows no differential transcription between males and females, while *cytb* is significantly more transcribed in males (Wilcoxon Rank-Sum test p-value < 0.001). x-axis: transcription level relative to the reference nuclear gene (*18S*).

**Figure 3:**
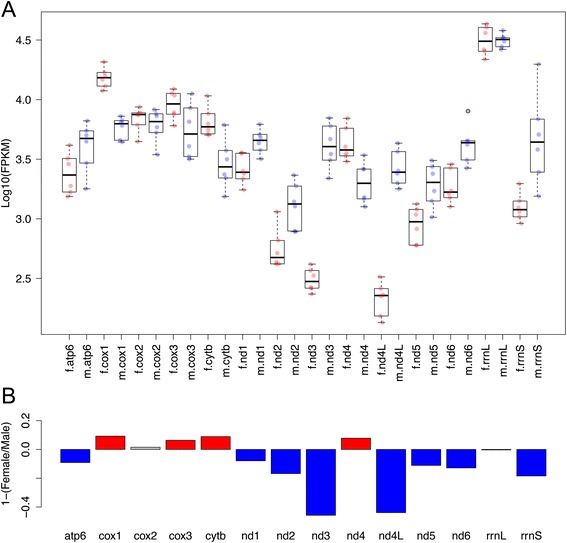
Transcription level of mitochondrial genes in mature gonads obtained by RNA-Seq. **(A)** Transcription levels expressed in Log10 (FPKM) of mitochondrial genes in females (red dots, “f.” prefix), and males (blue dots, “m.” prefix). **(B)** Barplot of 1-(Female transcription level/Male transcription level). 0 = no difference; positive values = transcription in females higher than in males; negative values = transcription in males higher than in females; red bars = transcription in females significantly higher than in males (Wilcoxon Rank-Sum test p-value < 0.01); blue bars = transcription in males significantly higher than in females (p-value < 0.01); grey bars = no significant differences. Data from [35].

The transcriptome of *R. philippinarum* mature gonads was already available [37], so we decided to perform a Real-Time qPCR analysis on individuals at the beginning of the reproductive season, to compare the two gametogenic stages. From observation of gonadic samples at optical microscope, out of the 32 analyzed specimens, 14 resulted to be females, and 18 males, both showing a prevalence of immature gametes. The transcription levels (relative to the reference gene *18S*) of *vasa*, a marker of germ line development [25,38], and *cytb* are reported in Figure 2. The *vasa* gene is transcribed with no significant difference between males and females, which is consistent with the two sexes being at a similar stage of gamete maturation. The transcription level of *cytb* in early gametogenic stage was significantly higher in males (Wilcoxon rank-sum test, p-value < 0.001), consistently with what recently described in other taxa [20,21]. RNA-Seq analysis [37] on late gametogenic stage (thus containing an abundance of mature gametes) highlighted an opposite situation, with *cytb* significantly more transcribed in females (p < 0.001, see Figure 3A,B). Furthermore, as discussed in the original paper, the female/male transcription ratios of the mitochondrially-encoded electron transport chain genes were variable: eight genes were significantly more transcribed in males, four in females, and two did not show significant differences (see Figure 3B).

Taken together, our data are consistent with the transcriptional activity in germ line mitochondria being variable between sexes and at different developmental stages. Moreover, we did not find evidence for transcriptional quiescence of mitochondria in female gametes.

### Mitochondrial membrane potential in *R. philippinarum* gametes

The presence of mitochondrial transcription is not evidence for respiration, so OXPHOS activity is usually assessed by the detection of mitochondrial membrane potential (Δψm). There are two ways to generate Δψm – via the respiratory chain and via reversal of the ATP synthase (e.g. [39]) and without measurements of O_2_ uptake it is nearly impossible to tell whether membrane potential is supported by fermentation or by OXPHOS. With this in mind, Δψm can be used as a proxy for OXPHOS activity as shown in eggs of several animal species (e.g.: Xenopus [40], mouse [41], zebrafish [42], and pig [43]), where an OXPHOS uncoupler (Carbonyl cyanide *p*-(trifluoromethyl) phenylhydrazone, FCCP) weakened/destroyed Δψm with consequent decrease/loss of mitochondrial dye fluorescence.

We stained *R. philippinarum* gonads with Mitotracker Green FM (revealing the whole mitochondrial mass) and Mitotracker Red CMXRos (sensitive to Δψm).

**Figure 4:**
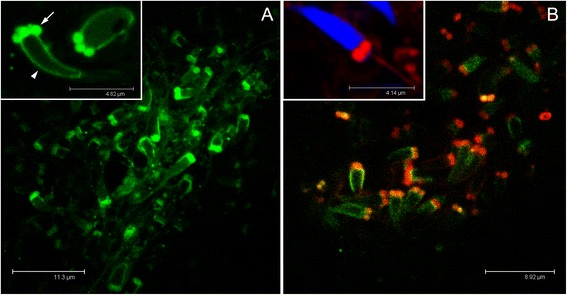
Mitochondrial inner membrane potential (Δψm) in spermatozoa. **(A)** The four/five mitochondria of the spermatozoon midpiece (arrow) are visible thanks to Mitotracker Green FM staining. The spermhead (arrowhead) is slightly rimmed by aspecific green staining. **(B)** The midpiece is strongly red-stained when treated also with Mitotracker Red CMXRos, indicating the presence of a high membrane potential. In the inset the spermhead is stained in blue (TO-PRO 3 nuclear dye).

The four/five mitochondria of the spermatozoon midpiece were easily recognizable using Mitotracker Green FM staining (Figure 4A), and the mitochondrial midpiece was also strongly red-stained when treated simultaneously with Mitotracker Green FM and Mitotracker Red CMXRos, indicating the presence of a high Δψm (Figure 4B). The result was confirmed using only Mitotracker Red CMXRos (inset in Figure 4B). In SMI species, the detection of Δψm in spermatozoa has been studied mainly for its possible implication in fertilization capability. The presence of Δψm in sperm was observed by fluorescent dyes in many animal species, among which cnidarians [20], insects [21,44], fishes [21,45], and several mammal species ([46], [47] and references therein). Of course, the implication for male-transmitted mtDNA viability was never taken into consideration, because of the absence of relevant paternal mitochondrial inheritance. Being *R. philippinarum* a DUI species, we expected to find some sort of quiescence or a limited activity of mitochondria in spermatozoa, as predicted for gametes that transmit mitochondrial genetic information to the progeny [18]. [37] speculated that sperm mitochondria in DUI species might use alternative pathways for energy production, like for example anaerobic metabolism, which bivalves are capable of (see [48] for a review). Such kind of metabolism could reduce ROS production, therefore eliminating at the root the problem of oxidative stress, but is an anaerobic mitochondrial metabolism compatible with the high Δψm we observed in sperm mitochondria, and with the energy demand for sperm swimming in a broadcast spawner? It has to be considered that differently from mammals, in which fertilization is internal and the survival of spermatozoa largely depends on extraneous nutrients provided by accessory secretions, in broadcast spawners fertilization takes place after the eggs and sperm have been shed into sea water, which is a poor source of organic nutrients. For example, unlike mammalian spermatozoa which in most species obtain their metabolic energy through glycolysis, sea-urchin spermatozoa appear to depend on strictly aerobic metabolism [49,50]. For these reasons, we think that the presence of mitochondrial aerobic respiration is a more parsimonious hypothesis, but detailed analyses of sperm energetic methabolism are necessary to address this issue.

**Figure 5:**
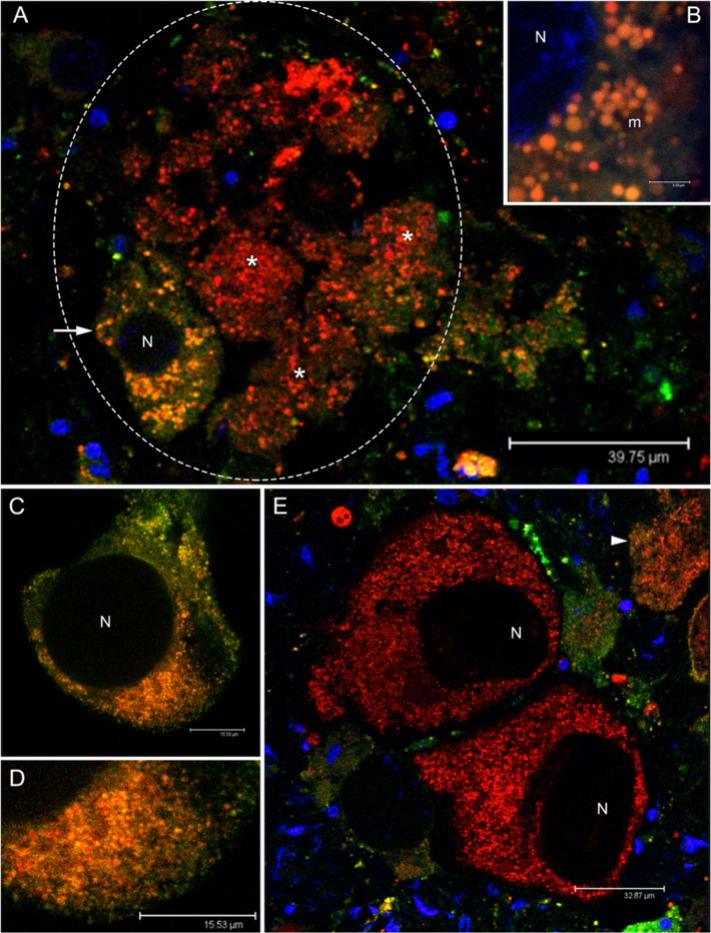
Mitochondrial inner membrane potential (Δψm) in oocytes (Mitotracker Green FM + Mitotracker Red CMXRos). **(A)** Female acinus (gonadic unit; dashed oval) containing several oocytes of which one of about 40 μm (arrow), positioned at the periphery of the acinus (acinus wall) and portions of oocytes of higher dimension (asterisks) in the acinus lumen. In the small oocyte the mitochondrial mass appears orange-stained, indicating the presence of a membrane potential (both the color components, Mitotracker Green FM and Mitotracker Red CMXRos, are present). The potential is higher in the biggest oocytes (asterisks), in which the mitochondrial mass is strongly red-stained (Mitotracker Red CMXRos component stronger than the Mitotracker Green FM one). Nuclei of cells around the acinus wall are visible in blue (TO-PRO 3 nuclear dye). **(B)** Detail of the mitochondrial mass of the small oocyte in **(A)**. Nucleus (N) lightly blue-stained. Mitochondria (m) in orange. **(C)** Oocyte of about 65 μm in which the mitochondrial mass appears orange-stained, indicating the presence of a membrane potential comparable to that of the 40 μm oocyte in **(A, B)**. The oocyte is starting the pedunculated stage. **(D)** Detail of the mitochondrial mass of the oocyte in **(C)**. The number of mitochondria appears largely increased in comparison to that of the 40 μm oocyte in **(A, B)**. **(E)** Two oocytes of about 100 μm at the pedunculated stage shortly before the release into the acinus lumen. The membrane potential is high, as indicated by the strong red staining of the mitochondrial mass (Mitotracker Red CMXRos component stronger than the Mitotracker Green FM one). At the periphery of a close acinus, a small oocyte is visible (arrowhead), showing green-orange staining, indicating the presence of a lower membrane potential in comparison to nearly mature eggs. Nuclei of surrounding cells are blue-stained. Nuclei of oocytes (N) are not always stained since the nuclear labelling is not easily absorbed through the yolk.

**Figure 6:**
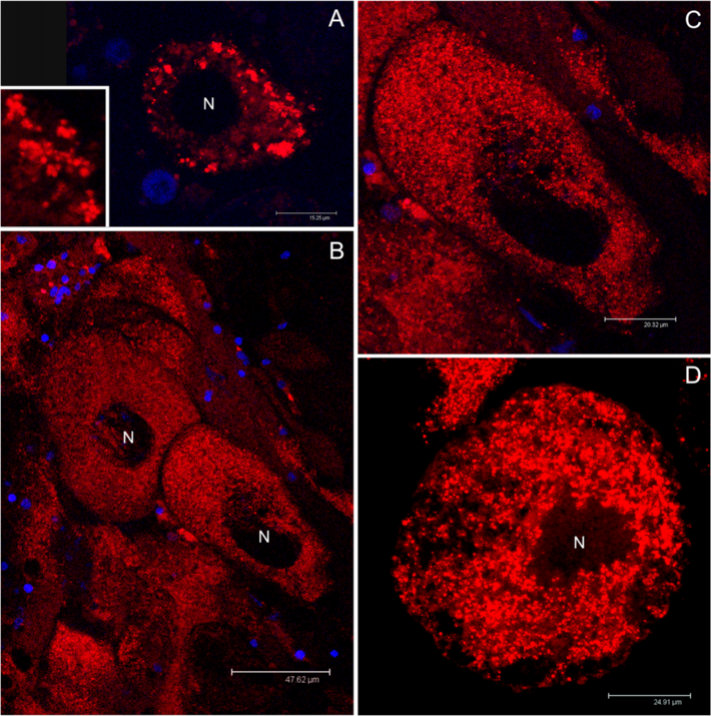
Mitochondrial inner membrane potential (Δψm) in oocytes (Mitotracker Red CMXRos). **(A)** Small oocytes show a less proportion of red-stained mitochondria, than **(B)** mature eggs (around 100 μm), in which all the mitochondrial mass is red-stained pointing to an overall high membrane potential. **(C)** Magnification of a mature oocyte inside the acinus (from B). **(D)** Spawned eggs as well show an overall high mitochondrial membrane potential. Nuclei of cells surrounding the acinus are visible in blue (TO-PRO 3 nuclear dye).

Female gonads showed acini containing several oocytes among which the smallest (about 40 μm), positioned at the periphery of the acinus, showed an orange-stained mitochondrial mass, indicating the presence of Δψm (both the color components, Mitotracker Green FM and Mitotracker Red CMXRos, are present giving an orange-staining) (Figure 5A,B). Bigger oocytes (about 65 μm) showed an orange-stained mitochondrial mass as well (Figure 5C), indicating the presence of a Δψm comparable to that of the 40 μm oocyte, but the number of mitochondria appeared largely increased (compare Figure 5B and D). Δψm was higher in bigger oocytes (about 75 μm) located in the acinus lumen, in which the mitochondrial mass is strongly red-stained (Mitotracker Red CMXRos signal exceeding the Mitotracker Green FM one) (Figure 5E). Also using only Mitotracker Red CMXRos, small oocytes (around 40 μm) showed a less proportion of red-stained mitochondria (Figure 6A), while in mature (around 100 μm) and spawned eggs, the mitochondrial mass was red-stained pointing to an overall high Δψm (Figure 6B-D). Summarising, female gametes at early gametogenic stages have fewer mitochondria with a lower Δψm in comparison with larger and mature oocytes. Both the mitochondrial mass and Δψm appear to increase during oocyte maturation (Figures 5 and 6). Accordingly, female germ line mitochondria do not remain quiescent during development, as they show both transcriptional activity and the presence of Δψm.

Our observations that mitochondria are active in both male and female gametes of *R. philippinarum* open the possibility that, rather than a selective silencing of organelles that need to function as genetic templates (eggs in SMI species, eggs and sperm in DUI species), some other solutions for the preservation of viable mtDNA may have evolved. Below, we report evidence for mitochondrial activity in eggs from the existing literature.

### Activity of oocyte mitochondria in Metazoa

Even if not designed specifically to detect mitochondrial OXPHOS activity, many studies that used Δψm-dependent staining on different animal species in several oocyte developmental stages are available in literature, thus allowing an overview on the activity of mitochondria in female germ line (see Table 1). Mitochondria show remarkable difference in morphology and metabolism between stem cells and differentiated cells. During the differentiation process from PGCs to mature oocytes, mitochondria develop complex cristae, an electron-dense matrix, and their Δψm increase significantly, as the cells shift from glycolysis to OXPHOS as principal pathway for ATP production [36,41,51-54]. Studies on different taxa also revealed that an increase of Δψm is necessary for oocyte maturation and for embryo developmental competence [52,53,55,56]. A reduction in mitochondrial bioenergetic capacity results in compromised fertilization and early embryo development, and is associated with meiotic spindle disorders that lead to defective chromosomal segregation. A decrease in Δψm also leads to the degradation of mitochondria in oocytes of women in advanced reproductive age (oocyte ageing [52]). On the contrary, oocytes with higher Δψm produce a higher rate of successful fertilization and embryo development [41,43,52,53,57], and the intracytoplasmic injection of viable mitochondria in presence of mitochondrial disfunction restores the normal conditions and inhibits oocyte fragmentation (see [51] and references therein). Furthermore, an elevated energy supply during late oogenesis is required for nuclear maturation and cytoplasmic remodeling [52,53] and the presence of Δψm is necessary for both calcium ion sequestration and the transport of protein precursors into mitochondria [55,56].

**Table 1:** **Detection of mitochondrial membrane potential (Δψm) in animal oocytes**

**Animal**	**Δψm-sensitive mitochondrial staining**	**Cell type and stage (st)**	**Bb**	**Ref.**
Fruit fly	MitoTracker Red CMX Ros (Molecular Probes) (M-7512)	nurse cell ec-st 6		Figure 2B in [105]
Zebrafish	MitoTracker Red 580 (Molecular Probes) (M-22425)	oocyte st 1	x	Figure 2G in [106]
	MitoTracker Red CMXRos (M-7512)	oocyte st 1	x	Figures 1, 2, 3 and 4 in [42]
	JC-1* (Cell Technology Inc.)	oocyte st 1	x	Figures 1, 2, 3 and 4 in [42]
African clawed frog	MitoTracker Red	oocyte st 2	x	Figure 1 in [40]
	JC-1	oocyte st 2	x	Figure 1 in [40]
Mouse	MitoTracker Orange (Invitrogen Ltd.)	oocyte st GV / GVBD		Figures 2, 3, 4, 5 and 6 in [107]
	JC-1 (Molecular Probes)	oocyte st MII		Figures 1,2 in [41]
Dog	MitoTracker Red CMX Ros (Molecular Probes) (M-7512)	oocyte st non-m / Ov / IVM		Figure 1 in [108]
Pig	JC-1 (Invitrogen)	oocyte st MII		Figure 1A in [43]
Dromedary camel	MitoTracker Orange CMTMRos (Molecular Probes) (M-7510)	oocyte st GV / MI / MII		Figures 2,3 in [66]
Human	JC-1 (Molecular Probes)	oocyte st GV / MI / MII		Figs 1, 2 and 3 in [57]

NOTE - Bb: Balbiani body staining/localization; ec: egg chamber; GV: immature oocyte, in which the mitochondria are aggregated around the germinal vesicle; GVBD: once oocyte maturation commences the GV breaks down and the mitochondria are seen to disperse throughout the cytoplasm during maturation, resulting in mitochondria occupying most of the egg volume; MII: metaphase II; Ov: ovulated = *in vivo* maturation; non-m: non-matured; IVM: *in vitro* maturation; *5,5′,6,6′-Tetrachloro-1,1′,3,3′-tetraethyl-benzimidazolylcarbocyanine iodide.

Although being clearly variable with stages, mitochondrial activity seems to be a fundamental determinant of competence in mature oocytes and early embryos. A possible criticism to this statement could be that not all the mitochondria in the oocyte need to be active in order to participate to the processes described above, and that a subpopulation of inactive organelles, namely those that will eventually end up in the PGCs, could be exempted from performing respiration in order to avoid ROS damage, as suggested by the “division of labour” hypothesis [19,20]. We address this point in detail in the discussion about selection mechanisms acting on germ line mitochondria.

### Is ROS production the root of all evil?

In aerobic organisms the production of ROS is inevitable and the bulk of oxidative pathways takes place in mitochondria (see [11] for a review). It is well known that ROS can damage cellular macromolecules, especially lipids, nucleic acids and proteins, and that at high concentration they are responsible for oxidative stress that can lead to cell death. On the other hand, physiological levels of ROS do not alter the integrity of cellular macromolecules [58], and ROS are actually essential for some cell functions and signalling pathways [11,56,59-65]. More specifically, ROS regulate cell cycle progression, microtubule organization, cell plate formation, nuclear division and migration, and a fine-tuned balance between ROS production and antioxidant activity is fundamental for the formation of competent oocytes and for normal growth and development [62,63]. In fact, ROS can switch the status of stem cells from proliferation to differentiation, and this role is conserved between animals and plants [59]; strikingly, [61] found that neural stem cells in human and mouse maintain a high endogenous ROS levels, and that the manipulation of such levels affects self-renewal and neurogenesis. A recent study on *Camelus dromedarius* oocytes [66] showed the presence of both mitochondrial membrane potential and colocalizing ROS, changing in relation with oocyte meiotic stage. Specifically, MitoTracker Orange CMTM Ros fluorescence intensity analysis revealed an increase of Δψm in oocytes during their maturation. That said, the source and the possible function of ROS in this process are not clear, and a burst of ROS during oocyte development could be just a side effect.

The free radical theory of ageing [12] has been criticized mostly for the lack of unambiguous evidence supporting a causal relationship between metabolic rate, ROS production and mtDNA mutation (see for example [13,67], and [68] and references therein). Such relationship is far from being clear, for example experiments on mice showed that mtDNA mutations are involved in the ageing process [69], but not in increased ROS production [68,70]. Also, experiments with dietary supplements, failed to demonstrate a role of antioxidants in increasing lifespan; on the contrary, they caused lifespan reduction and mortality increase [68]. There is evidence that metabolic rate is unrelated to longevity (see for example [71]), supporting the dependence of mtDNA somatic mutations from processes unrelated with energy production. Recently, the contribution of oxidative stress in somatic mtDNA mutations was investigated in human brain cells [72] and in *Drosophila* [73]. Both the studies argue against free radical theories of ageing, and suggest that mtDNA replication is responsible for the bulk of accumulating point mutations in mtDNA (for a discussion on the possible role of replication in germ cell mtDNA mutation see [37]). In other words, it is not correct to assume that usage necessarily leads to more ROS leak, but it might nonetheless be true that usage contributes to a higher mutation rate as a result of greater mtDNA turnover and copying errors.

Another major point to take into consideration when evaluating the role of ROS production in mtDNA damage, is that cells have an impressive repertoire of antioxidant defenses, and that oxidative stress mainly results from the disruption of the equilibrium between ROS production and scavenging [11,13,56,68,74]. Mitochondrial antioxidant enzymes are associated with mtDNA [75] as integral constituents of the nucleoid, a set of DNA-binding proteins involved, for example, in mtDNA maintenance, protection, and transcription [76,77]. The role of antioxidant enzymes and nucleoid proteins in preserving mtDNA from oxidative damage deserves further investigation. An increased production of such molecules during germ line development could be a mechanism for the preservation of mitochondrial genetic information.

Finally, from a methodological point of view, several works state that many precautions must be taken in experimental set up (reviewed in [78]), since ROS can be underestimated or not detected [60], and some ROS detection methods can be affected by pH changes (see for example [78] and [79]). The complexity of the issue is proved by the debate on highly specialized journals, with ongoing discussion about the importance of establishing standards for methods used in the field, and how results should be interpreted [80]. Even if mitochondria are a major source of ROS, many radicals of other origin are present in the cytoplasm, and must be considered when assessing ROS production in cells. Also, the extremely short lifetime of reactive species [81] and the variety of existent antioxidant systems that are able to capture these species make them difficult to be detected.

### How is the inheritance of a viable mitochondrial genetic information achieved?

As highlighted in the former section, ROS may not be the main cause of mtDNA damage, but given the high mutation rate of mtDNA and the absence of sexual reproduction, mitochondria still have to cope with Müller’s ratchet, so there must be a mechanism to avoid the transmission of damaged organelles through generations. Despite the high mtDNA copy number in mature oocytes (about 100,000 copies in mammals), mtDNA sequence variants segregate quickly between generations, suggesting the existence of a mitochondrial bottleneck [82,83]. Such bottleneck favors the transmission of a homoplasmic population of mtDNA [15,36,83], but the random segregation of mtDNA variants cannot explain the observed bias against the transmission of deleterious mutations [83-88], or the strict segregation of specific mitochondrial lineages in a naturally heteroplasmic system [29], which can both be explained by the existence of a concurrent selective process. But how does selection work? And when does it occur?

In animal oocytes, the mitochondria that participate in germ plasm formation are highly active ([40] and Table 1), and form an evolutionarily conserved structure, the Balbiani body (Bb), that was observed in developing oocytes of many animals [25,89,90]. A role of the Bb in the selection of functional mitochondria for PGCs was hypothesized by many Authors [89-94]: in particular, it was proposed that mitochondria with high inner membrane potential tend to be recruited preferentially into the Bb, that would be responsible for the preferential transmission of wild-type mtDNA [95]. Accordingly, [42] observed that mitochondria displaying higher Δψm are recruited into the Bb. Interestingly, the so called “nuage” material present in the Bb includes molecules involved in mitochondrial genome preservation [96]. Therefore, the close relationship of mitochondria with germ plasm constituents during early gametogenesis may also be related to the preservation of mtDNA integrity [42].

Other than Δψm, another mitochondrial phenotype that was proposed to be under selection in the germ line is mtDNA replication [88]. According to this hypothesis, mtDNA replication is coupled with mitochondrial activity and is dependent on mitochondrial fitness: [88] showed that impaired replication of mutant genomes during *Drosophila* oogenesis prevents the transmission of mutant mtDNAs which are purged from the mitochondrial population. Organelles containing a high proportion of wild-type mtDNA would replicate faster than those containing more mutant genomes, leading to a decrease in the proportion of mutant mtDNA. Selective replication of mtDNA variants has been proposed also in mouse ovarian follicles [97], suggesting that replicative competition could also be involved in purifying selection of mtDNA in mammals. On the other hand, also mtDNA carrying deleterious mutations can have a clear replicative advantage over the wild-type mtDNA. One well studied example is the “petite” mutation in *Saccharomyces cerevisiae*, which entails a respiratory chain deficiency due to a mtDNA deletion [98]. Similar mitochondrial aberrancies in the germ line could have a proliferative advantage, but they probably would not be selected depending on their Δψm, or the consequence for gametes may be inactivity and unsuccessful fertilization. That said, some deleterious mtDNA mutants that confer a replicative advantage may not cause big enough functional impairments, so the selection would be unable to purge them (e.g. MELAS-associated mutation, [99]).

The recruitment of healthy mitochondria into the Bb and the selection on mtDNA replication, could act in concert, strengthening the selective process on mtDNA in germ line [88].

### Mitochondrial checkup

The existence of purifying selection in the female germ line has been so far suggested by multiple studies (reviewed in [83]), but the mode and tempo of action are still unclear. Also the level at which the selection acts is a matter of debate: we propose that, each generation, mitochondria undergo an “extensive checkup” from early gametogenesis to late embryo development. During these phases, mtDNA and mitochondrial functions are exposed to selection at different levels, from subcellular, to cellular, to organismal (Table 2). One type of selection would act at the organelle level, during gametogenesis, by means of the functional readout of mtDNA within the mitochondrion (by both OXPHOS activity and replication [86,88]). Mutant mitochondria would be either degraded by mitophagy (sequestration of mitochondria by autophagosomes followed by degradation in lysosomes [83]), or outcompeted by wild-type organelles. Most active mitochondria would then be recruited in the Bb. A selection at the cellular level would occur during gametogenesis, acting mostly on processes involved in gamete proliferation (chromosomal segregation, cell division, cytokinesis) and maturation, but also during the fertilization process; mtDNA mutations causing unfit cells/gametes would be eliminated by cell apoptosis [85] or by unsuccessful fertilization. This type of selective process is consistent with the observation that only a very small number of primary oocytes reach maturation, and only a very small percentage of gametes achieve a successful fertilization [52,100]. A third type of selection would act at the organismal level, after the formation of the zygote, during early development; mitochondria play a central role also in this phase, so deleterious mutations will be purged through the selective survival of fittest embryos. Reproduction is a quite inefficient process, and not only in organisms that produce billions of gametes (e.g. broadcast spawners). For example, as [52] pointed out, in humans over half of normally fertilized oocytes will never progress to birth, and the frequency increases with the age of the parents. Such a low success rate, however, makes evolutionary sense, because it is the mechanism by which natural selection get rid of deleterious mutations, and because mitochondria have a pivotal role in gametogenesis and development, the strongest selection on mitochondrial functions occurs at these stages. We also want to point out that if, as suggested by the cited literature, there is a selection acting on mitochondrial functions (e.g. OXPHOS and replication), inactive mitochondria could not be screened by such selective process. This also means that if after the selection of PGC mitochondria by the Bb these organelles will not participate actively to early embryo development, only somatic mitochondria will be subject to the selective process at the organismal level, and germ line mitochondria would undergo selection only during gametogenesis, that is the phase in which the selection is stronger due to the low mtDNA copy number per mitochondrion. Indeed, in the presence of high mtDNA copy number per organelle, wild-type functional copies of the genes can complement nonfunctioning copies, preventing the expression of a mutant phenotype, thus weakening the action of purifying selection, but a low mtDNA copy number per organelle exposes deleterious mutations to a stronger selection [37,83,86,88]. Such selection does not operate only on mtDNA: for example, from the zygote stage on, it targets both the mtDNA and the nuclear genes involved in OXPHOS and other mitochondrial functions: as mitochondria have to function in a newly formed nuclear background, mitonuclear incompatibilities are responsible for a substantial percentage of developmental failures [15].

**Table 2:** **Selection on mitochondrial functions**

**Stage**	**Level**	**Mechanism**
Gametogenesis	organelle	autophagy of unfit mitochondria selection of high Δψm mitochondria (Bb) replication advantage of wild-type mtDNA
Gametogenesis Fertilizing gametes	cell	apoptosis of unfit cells fertilization failure
Zygote Early embryo	organism	development failure

Note: the targets of selection are mtDNA and/or nuclear genes for mitochondrial function.

## Conclusions

Given the peculiar features of mitochondrial inheritance, genetics and physiology, how transmission of viable mtDNA through generations is achieved is object of debate. A proposed theory was the “division of labour” between male and female gametes, according to which mitochondria that represent genetic templates for the progeny are inactive, in order to preserve them from oxidative damage. On the contrary, mitochondria that need to perform the energetic function (i.e. sperm mitochondria) are not inherited. However, in DUI animals both gamete types transmit their mitochondria to the next generation, so we tested whether the mitochondrial activity was approximately the same in eggs and sperm, using the same methodologies recently utilized in other animals. Our data support this hypothesis, leading to the question: how do DUI gametes produce the energy level necessary for fertilization and embryo formation? How do DUI animals cope with the OXPHOS-induced mitochondrial damage in the gametes? One could expect, and we are going to test it, that antioxidant enzymes are upregulated in gametes of DUI animals compared with animals with maternally inherited mtDNA. However, our findings and the literature we discussed may be consistent with an alternative hypothesis: template mitochondria are not functionally silenced, on the contrary their activity might be fundamental for the inheritance mechanism. We think that during gametogenesis, fertilization and embryo development, mitochondria undergo selection for different traits (e.g. replication, Δψm), increasing the probability of transmission of functional organelles. In these phases of life cycle, the great reduction in mtDNA copy number per organelle/cell and the stochastic segregation of mtDNA variants would greatly improve the efficiency of selection. When a higher mtDNA copy number per organelle/cell is present, selection on mtDNA deleterious mutants is less effective, due to the buffering effect of wild-type variants. In our opinion, a combination of drift and selection on germ line mtDNA population, might be responsible for the maintenance of viable mitochondrial genetic information through generations, and a mitochondrial activity would be necessary for the selective process. Of course, mutations can escape the proposed “mitochondrial checkup” if their phenotypic effect is either mild or not expressed in the context in which selection takes place.

The metabolic activity of mitochondria undergoes dramatic changes during gamete maturation in many species. This complexity together with known methodological difficulties can be the explanation of different results in assessing mitochondrial activity and the effects of ROS on mtDNA damage. For these reasons, and given the biological and evolutionary relevance of the discussed mechanisms, it will be important to perform in depth analyses on different developmental stages of a widespread variety of taxa. For example, a better understanding of the dynamics of ROS production and scavenging in the germ line would greatly improve our knowledge about the processes of cellular senescence and molecular degradation, and the related protective mechanisms that eukaryotes have evolved. The investigation of the discussed selective processes and the role of the Bb, which are strictly related to mitochondrial performances, namely replication and the production and maintenance of Δψm, would also be of great importance. In this concern, the exploitation of the DUI system could be helpful, since in DUI males the mitochondria that end up into germ cells derive exclusively from the mitochondria of the fertilizing spermatozoon and we can study their activity all throughout the developmental stages, from male gamete formation to fertilization, and follow their migration during male embryo development.

## Methods

### Sampling

Specimens of the DUI species *Ruditapes philippinarum* (Manila clam) were sampled in Goro (Italy), thanks to the collaboration with the CRiM mollusc research center (http://www.istitutodelta.it/inglese/index.htm). Gonadic tissues were excised from adult specimens for which sex and developmental stage will be carefully assessed by examination at optical microscope. Samples were immediately processed. Spawning induction was performed as in [26].

### Transmission electron microscopy (TEM)

Sample were prepared using a protocol optimized for preparation of marine animals [25,26]. Gonadic tissue was dissected with a scalpel and immediately fixed for 2 hours in glutaraldheyde 2%, then rinsed in salt water and post-fixed in 1% osmium tetroxide in salt water for 1 h. After rinsing, we proceeded with dehydration with increasing concentrations of acetone (50–100; 70% acetone solution contained 1% uranyl acetate). Samples were then embedded in resin (Fluka Durcupan ACM). Polymerization was completed after 3–4 days at 60°C, followed by 1–2 days at 45°C. A Reichert ultramicrotome was used to cut silver-gold ultrathin sections (60–90 nm) using diamond knives. Ultrathin sections were collected on 75 mesh, copper grids coated by formvar and then stained with 3% uranyl acetate and with lead citrate. Sections were examined using a Philips CM100 (PW6021) TEM at 80 kV. Pictures were recorded with a Kodak MEGAPLUS Camera, Model 1.6i, using AnalySIS® Software.

### Real-time qPCR

Total RNA was extracted from gonads of 32 adult gametogenic specimens (prevalence of immature gametes) using TRIzol RNA Isolation Reagent (Life Technologies). The quality of RNA was assessed by gel electrophoresis and quantified with a Nanodrop ND-1000 spectrophotometer. cDNA was synthesized from diluted RNA using high Capacity cDNA Reverse transcription Kits (Invitrogen) following manufacturer’s indications. cDNA samples were then checked with Nanodrop ND-1000 and kept at −20°C until utilization. A Real-Time qPCR analysis was performed on cDNA with a StepOnePlus system (Applied Biosystems), following the protocol described in [101]. The nuclear ribosomal gene *18S* was chosen as endogenous control, the *vasa* gene was chosen as a marker of germ line activity, and the cytochrome b (*cytb*) genes (M-type in male gonads, F-type in female gonads) were used to assess the transcriptional activity of mitochondria. Specific primers were designed with Primer3 ([102]; see [101] for other details). Each reaction was performed in duplicates, for a total number of 256 reactions (32 specimens × 4 genes × 2 technical replicates). The ΔΔCq quantification method [103] was used to determine the relative amount of targets in each sample. Plots and statistical analysis were performed using R v3.1.1.

### Mitochondrial Δψm detection

Fluorescent dyes were used to visualize the activity of mitochondria in gonadic tissue of males and females. Using a fluorescent dye that stains the mitochondrial mass regardless of its activity (Mitotracker Green FM, M-7514, excitation⁄emission 490⁄516 nm, Molecular Probes [60,104]), we visualized all the mitochondria present in the gametes. Using another fluorescent dye (Mitotracker Red CMXRos, M-7512, excitation⁄emission 579⁄599 nm, Molecular Probes) that is imported into active mitochondria proportionally to their membrane potential [60,104], the organelles with an active OXPHOS were visualized. Performing a simultaneous staining, the co-localization of the two combined Mitotracker dyes (green and red) showed active mitochondria in a yellow/orange color. In contrast, mitochondria with low or absent Δψm remained green, as Mitotracker Green FM does not respond to Δψm. Experiments were optimized taking into consideration the physiological conditions of a marine species, so either PBS or saltwater solutions were utilized. The vital mitochondrial dyes were dissolved in anhydrous dimethylsulfoxide (DMSO) (Sigma) and added to PBS (or saltwater) at 400 and 500 nM (final concentration, Green FM and Red CMXRos, respectively). The solutions were added with TO-PRO 3 nuclear dye (Molecular Probes) (final concentration 500 nM). Gonadic samples were incubated in these solutions for 40 minutes at room temperature in the dark. Then, samples were washed once in PBS (or saltwater), put on slides, and mounted in 2.5% 1,4-diazabicyclo [2.2.2] octane (DABCO; Sigma), 50 mM Tris (pH 8) and 90% glycerol. Samples were visualized by a Leica SP2 confocal microscope. Even though both PBS and saltwater gave a positive staining of mitochondria, the experiments performed using saltwater resulted in a cleaner signal (probably due to a better absorption of the fluorescent dyes).

## Reviewers’ comments and response

### Reviewer #1: professor Nick Lane

This is an interesting and worthwhile paper that addresses an important subject, although I find a number of the conclusions and interpretations to be too sweeping for my comfort. I have to say I wouldn’t immediately revise my current views in light of the data presented here.

The data in the paper seem reasonably sound to me, though there is again scope to question some of the interpretations (see below). On the other hand, the collected data from other studies are not easy to generalise, and in my view there is too much conflict there to really support the conclusions drawn. But none of that should prevent publication of a worthwhile contribution to the literature.

Authors’ response:* First of all we want to thank Prof. Lane for his detailed and insightful review of the manuscript. Our first goal when we decided to submit this work to Biology Direct was to stimulate a scientific debate about one fundamental topic in biology such as mitochondrial inheritance, so we greatly appreciated Prof. Lane’s point of view.*

*With this paper we wanted to propose an (in our opinion reasonable) alternative hypothesis to the “division of labour” theory, given our results and observations on other animals.*

*We changed some parts of the text following Prof. Lane’s comments.*

My major concern is with how generalisable the conclusions can be from the unusual case of doubly uniparental inheritance in bivalves; and a lack of context for the interesting natural history of mtDNA in these mussels, which the authors plainly know a great deal about from their earlier publications. Yet some important details are not addressed here at all:

Authors’ response:* Despite many aspects of DUI are still unknown, it seems reasonable to assume that it evolved as a modification of strictly maternal inheritance (SMI). There is evidence consistent with this assumption: i) the scattered phylogenetic distribution of DUI among bivalves indicates that DUI can revert into SMI (see for example: Breton et al. 2007, Theologidis et al. 2008, Zouros 2013); ii) Mytilus hybrids revert to SMI (Kenchington et al. 2009); iii) the ancestral condition in molluscs appears to be SMI, while DUI is a derived character (either monophyletic or polyphyletic). Thus it is parsimonious to assume that DUI has derived from SMI by modification of the molecular machinery of SMI, and it is a common opinion among the researchers working on DUI that this unusual system can be an useful model to study basic issues about mitochondria (see for example: Passamnti e Ghiselli 2009 and Zouros 2013). About the lack of context for the natural history of mtDNA in these bivalves, DUI is a very complex system, so it was not possible to address all the details about its evolutionary patterns. We clarified in the manuscript some features that are important to understand our conclusions, and we also addressed some others below. Of course it was not possible to provide an exhaustive overview, but we think that the discussion points raised by the Reviewers were all covered.*

*References:*

*Breton S, Beaupre HD, Stewart DT, Hoeh WR, Blier PU: The unusual system of doubly uniparental inheritance of mtDNA: isn’t one enough? Trends Genet 2007, 23: 465–474.*

*Kenchington EL, Hamilton L, Cogswell A, Zouros E: Paternal mtDNA and maleness are co-inherited but not causally linked in mytilid mussels. PLoS ONE 2009, 4: e6976.*

*Passamonti M, Ghiselli F: Doubly uniparental inheritance: two mitochondrial genomes, on precious model for organelle DNA inheritance and evolution. DNA Cell Biol 2009, 28:79–89.*

*Theologidis I, Fodelianakis S, Gaspar MB, Zouros E: Doubly uniparental inheritance (DUI) of mitochondrial DNA inDonax trunculus(Bivalvia: Donacidae) and the problem of its sporadic detection in Bivalvia. Evolution Int J Org Evolution 2008, 62: 959–970.*

*Zouros E: Biparental Inheritance Through Uniparental Transmission: The Doubly Uniparental Inheritance (DUI) of Mitochondrial DNA. Evol Biol 2013, 40: 1–31.*

what is the evolution rate of mtDNA in bivalve mussels compared with other animals? My guess is that it would be quite low compared with other metazoans, given their sessile lifestyle, in adulthood at least. I don’t know much about the behaviour of juvenile forms. Our own modelling work (Hadjivasiliou et al. 2013) suggests that BPI or male mtDNA leakage might be relatively common in organisms with low mitochondrial number and low mtDNA mutation rates, and it can be very ambiguous whether inheritance in BPI or UPI in e.g. sponges and corals. In other words, if the mutation rate is low, there is less, if any, need to repress it further by transcriptional inactivation.

Authors’ response:* A very high DNA polymorphism in bivalves is known to date (Saavedra and Bachere 2006), and was recently confirmed by the whole genome sequencing of the Pacific oyster (Zhang et al. 2012). The evolution rate of mtDNA in bivalves is high, and DUI mt genomes evolve faster than other invertebrates and vertebrates (Breton et al. 2007, Gissi et al. 2008, Zouros 2013 and references therein).*

*We also would like to point out that the model elaborated in Hadjivasiliou et al. 2013 compared biparental inheritance and uniparental inheritance. DUI is neither, so we think that the conclusions might not apply to a doubly uniparental system (it would be actually very interesting to build a mathematical model for DUI).*

*References:*

*Breton S, Beaupre HD, Stewart DT, Hoeh WR, Blier PU: The unusual system of doubly uniparental inheritance of mtDNA: isn’t one enough? Trends Genet 2007; 23: 465–474.*

*Gissi C, Iannelli F, Pesole G: Evolution of the mitochondrial genome of Metazoa as exemplified by comparison of congeneric species. Heredity 2008, 101: 301–320.*

*Hadjivasiliou Z, Lane N, Seymour M, Pomiankowski A: Dynamics of mitochondrial inheritance in the evolution of binary mating types and two sexes. Proc Biol Sci 2013, 280: 20131920.*

*Saavedra C, Bachere E: Bivalve genomics. Aquaculture 2006, 256: 1–14.*

*Zhang G, Fang X, Guo X, Li L, Luo R, Xu F, et al.: The oyster genome reveals stress adaptation and complexity of shell formation. Nature 2012, 490: 49–54.*

*Zouros E: Biparental Inheritance Through Uniparental Transmission: The Doubly Uniparental Inheritance (DUI) of Mitochondrial DNA. Evol Biol 2013, 40: 1–31.*

The authors note that there can be up to 50% disparity in sequence between male and female mtDNA, an extraordinary difference. It’s hard to imagine how a single nuclear genome can be equally well coadapted to both nuclear genomes; are there multiple isoforms of nuclear-encoded respiratory proteins?

Authors’ response:* Unfortunately, up to now no genome of DUI species is available, and the transcriptomes sequenced so far were not analysed for alternative splicing/isoforms. We are working right now to collect new data and, as far as we know, also other Labs working on DUI are doing the same. More information will come about this interesting topic. The existence of isoforms for nuclear-encoded respiratory proteins is actually one of the most popular hypothesis to explain how DUI organisms can cope with mtDNA heteroplasmy.*

The work of Pierre Blier, Donald Stewart and others (including the authors) suggests that the mutation rate of male mtDNA is substantially faster than female mtDNA, implying that there are indeed different constraints on male vs female mtDNA. This is made more striking by periodic role reversals – the ‘masculinization’ of female somatic mtDNA, which is recombined onto the male gonadal targeting sequence, enabling the replacement of male gonadal mtDNA with female mtDNA (e.g. Breton et al. 2007). My understanding is that fitness (such as capacity for swimming) improves after such masculinisation events, implying that either coadaptation to the nuclear background improves, or that male mtDNA mutations are eliminated by these periodic take-overs. It is therefore not strictly true to argue that bivalves are an evolutionary stable system. Are such periodic role reversals necessary or just beneficial? Freshwater molluscs don’t appear to undergo such mascularizations; what is their mtDNA mutation rate in males and females then? Given these specific issues relating to dUPI in bivalves, I think it is unreasonable to extrapolate to other systems as if these factors did not exist.

Authors’ response:* The idea that masculinization (or ‘role reversal’) is a widespread phenomenon in DUI arose to explain its scattered phylogenetic distribution in bivalves: indeed, the hypothesis of a single origin of DUI (with the subsequent loss in some species) requires the assumption of masculinization events in several branches of the bivalve tree (see: Theologidis et al. 2008 and Zouros 2013 for a detailed discussion). Recent analyses may suggest the involvement of selfish elements (i.e. viral sequence endogenization in the M-type mt genome) in the transmission of sperm mitochondria in DUI (Milani et al. 2013, Milani et al. 2014). According to this, a multiple origin of DUI may be also taken into account, so there would be no need of multiple role-reversal events to explain its phylogeny.*

*Furthermore, masculinization has been observed only in hybrids between Mytilus species (M. edulis/M. galloprovincialis) and in some european populations of M. trossulus, so it cannot be considered a rule, but rather an exception. Masculinization sets to zero the divergence between the two differently transmitted mt genomes, so recently masculinized genomes show a lower divergence with the F genome (around 2%), compared with the divergence between F and M (around 25-30% in Mytilus). The F/M divergence observed in other DUI groups is even higher that the divergence observed in the Mytilus edulis complex [for example 34% in R. philippinarum (family Veneridae), and up to 50% in freshwater mussels (family Unionidae)]. In the phylogeny of unionids (the other family, other than Mytilids, in which DUI has been extensively studied) the F mt genomes from all the species, genera and subfamilies cluster together, and so do the M genomes (gender-joining pattern); the occurrence of role-reversal events in unionids cannot be ruled out, but the non-fixation of masculinized mt genomes is almost certain (Zouros 2013).*

*DUI is an evolutionary stable mechanism, F and M genomes being separated for million years (>200 million years in Unionids, see Breton et al. 2011; for a thorough discussion about these aspects of DUI, see Zouros 2013). We think that in such a long evolutionary time, nuDNA must have coevolved with both mtDNA types. M-type mtDNA evolves faster, but we do not think that this is because of a relaxed selection: M-type has to perform in very important contexts, such as gametogenesis and fertilization, so it has to be under selective constraints. Under this light, there is no need for role-reversal events to restore M-type viability and/or mito-nuclear match, and role-reversal is not needed for the existence of DUI. Supporting this viewpoint, a recent SNP analysis of the mitochondrial DNA population in R. philippinarum male and female gonads showed a lower amount of deleterious polymorphism in M-type (see Ghiselli et al. 2013 for a detailed discussion). Consistently, although we have not yet found any traces of masculinization events in R. philippinarum, this species is highly invasive, therefore we think that the absence (or paucity) of role-reversal events does not affect the evolutionary success of this organism.*

*A possible explanation of M-mtDNA faster evolution might be sperm competition and/or coevolution between M mitochondria and nuclear factors involved in sexual development, spermatogenesis and reproduction, which are known to experience accelerated evolution (also discussed in Ghiselli et al. 2013).*

*References:*

*Breton S, Ghiselli F, Passamonti M, Milani L, Stewart DT, Hoeh WR: Evidence for a Fourteenth mtDNA-Encoded Protein in the Female-Transmitted mtDNA of Marine Mussels (Bivalvia: Mytilidae). PLoS One 2011, 6: e19365.*

*Ghiselli F, Milani L, Guerra D, Chang PL, Breton S, Nuzhdin SV, et al.: Structure, transcription, and variability of metazoan mitochondrial genome: perspectives from an unusual mitochondrial inheritance system. Genome Biol Evol 2013, 5: 1535–1554.*

*Milani L, Ghiselli F, Guerra D, Breton S, Passamonti M: A comparative analysis of mitochondrial ORFans: new clues on their origin and role in species with doubly uniparental inheritance of mitochondria. Genome Biol Evol 2013, 5: 1408–1434.*

*Milani L, Ghiselli F, Maurizii MG, Nuzhdin SV, Passamonti M: Paternally transmitted mitochondria express a new gene of potential viral origin. Genome Biol Evol 2014, 6: 391–405.*

*Theologidis I, Fodelianakis S, Gaspar MB, Zouros E: Doubly uniparental inheritance (DUI) of mitochondrial DNA in Donax trunculus (Bivalvia: Donacidae) and the problem of its sporadic detection in Bivalvia. Evolution Int J Org Evolution 2008, 62: 959–970.*

*Zouros E: Biparental Inheritance Through Uniparental Transmission: The Doubly Uniparental Inheritance (DUI) of Mitochondrial DNA. Evol Biol 2013, 40: 1–31.*

Some specific comments in rough order of appearance.

Page 4, increase in ROS production in the free-radical theory of ageing. I’m not really aware of any strong evidence to suggest that ROS levels increase with age. This is a straw-man view of the free-radical theory. The work of Gustavo Barja, among others (eg. Barja 2012) has a far more nuanced and modern conception of the free-radical theory, incorporating factors such as signalling.

Authors’ response:* We agree about all the points raised here. We strengthened these concepts in the text, and we added the suggested reference.*

Page 8. Transcript levels of *cytb* – this is clearly lower in females in Figure 2, but the authors immediately contradict their own data with reference to an earlier paper. The transcript data generally is somewhat inconclusive, and it would be wise to include a warning that there is no necessary link between transcript levels and protein levels, especially in a mitochondrial system where the stoichiometry of subunits appears to be extensively controlled by post-translational modification.

Authors’ response:* We did not contradict our previous data. The analyses were performed at different stages of gametogenesis, and we pointed out in the manuscript that different stages show different mitochondrial activity. We rephrased the text in order to clarify it.*

Page 9 membrane potential. I’m not an expert on fluorescent dyes at all, but I am aware there are a number of issues relating to their use, concentration, exposure time and so on. How these compare between studies I don’t really know. I’m struck here that the merges often do not show orange (for both MitoTracker Green and MitoTracker Red), but straight red, implying an active membrane potential but insufficient MitoTracker Green to pinpoint the mitochondria. That looks like an issue of dose or exposure to me; if it is not, the authors should explain more clearly. I am aware that in the da Paula papers cited here, over-exposure of MitoTracker red did show a membrane potential in oocytes, hence dose and exposure are important. These details are not clear from the Methods, and certainly not in relation to other papers. The difference in positive staining with saline vs PBS mentioned in the Methods section also implies there were some issues with absorption and exposure.

Authors’ response:* We agree that there are a number of issues relating to the use of dyes (concentration, exposure time, etc.), as we stated in the manuscript. In our opinion it is quite difficult to say whether the fluorescence is present because of an excess of dye concentration/incubation time or it is not present because of insufficient dye concentration/incubation time.*

*In this work we tried to point out that comparisons can only be performed between comparable stages of development and that generalizations deriving from observations on a single stage should be avoided. This is why we analyzed different stages of gametogenesis.*

*In the Methods section we reported all details about the used methodologies, that followed the manufacturer protocol. That means that we used the dyes within the indicated concentration/timing range. Obviously it is impossible to use exactly the same protocol in every species, because of intrinsic differences (e.g.: different osmolarity, yolk storage, etc.). Protocol optimization is always necessary, but nonetheless, with caution, the results obtained can and need to be compared.*

*In our experiment MitoTracker® Green FM was sometimes weak, but we avoided to exceed in the concentration to prevent aspecificity (as suggested for this specific reagent).*

*We had no problem with MitoTracker® Red CMXRos, and, since it is specifically sequestered in mitochondria in relation to the presence of membrane potential, we think that the presence of Δψm is supported, also considering that in almost all the cited references different dyes showed the presence of membrane potential in oocytes.*

*Moreover, since in the same gonadic section oocytes showing different degrees of staining (different shades of yellow and red) are visible, we think that the signal did not reach saturation.*

Also relating to membrane potential, there are of course two ways to generate membrane potential – via the respiratory chain and via reversal of the ATP synthase. The fact that (in mammals at least) the inhibitor of ATPase IF1 is constitutively expressed, and knockdown has major effects on cristae morphology (e.g. see papers by Michael Duchen e.g. Faccenda et al. 2013) implies that reversal is common. Again, I have no idea whether this is also true in bivalves. Without measurements of O_2_ uptake it is nearly impossible to tell whether membrane potential is supported by fermentation or by oxphos. Therefore again I feel the conclusions are a bit too sweeping given the ambiguity of the data.

Authors’ response:* We agree. We modified the text to include other possible options for ATP production other than OXPHOS.*

The authors discuss critical differences in cristae morphology, transcription and membrane potential during different stages of female germline development. I am sympathetic to their view that there are periods when oocyte mitochondria appear to be active, but I am not convinced that they have proved it here. Moreover, it is feasible that mitochondrial activity is necessary at certain times and not at others, and that even partial repression for part of the time is better than no repression at all. Partial repression could lower the incidence of new mtDNA mutations, enabling selection to operate more effectively (by lowering heteroplasmy, so increasing the variance between mtDNA in oocytes) at certain time points only.

Authors’ response:* We think we provided information that cast doubt about a ‘universal’ functional silencing of oocyte mitochondria. Of course, as we stated in the manuscript, it will be fundamental to perform in depth analyses on different developmental stages of a widespread variety of taxa, in order to shed light on this point.*

Page 11–12 oocyte activity in Metazoa – much of the work cited in this section suffers from the same difficulties of interpretation discussed above. These are difficult studies and the conclusions here are maybe a bit too black and white.

Authors’ response:* Unfortunately, all the literature related to this topic suffers of the mentioned difficulties. We agree about these studies being difficult to perform and interpret, so we tried to take into consideration the different points of view and not to generalise. We modified our phrasing accordingly.*

Page 12–13 are ROS the root of all evil? I agree with much of this discussion, but again I think most people would. I don’t think it is true that ROS signalling is overlooked; there are entire journals devoted to the subject. There is indeed a general agreement that most mtDNA mutations are a result of copying errors rather than ROS leak, but I’m not convinced that this alters the argument for repression of replication and transcription. If female mtDNA acts as a template (I’m personally not convinced by this either) then it doesn’t matter whether mutations are caused by ROS or copying errors: greater use leads to higher mtDNA turnover, which leads to more errors.

Authors’ response:* We agree, and we integrated Prof. Lane’s comment in the text.*

It seems clear that in most bilaterians, the female germline is indeed repressed in relation to the male germline, and it might be the case that mtDNA transcription and replication is low in immature oocytes. Yet there is no agreement on whether there is a physical mitochondrial bottleneck or not (and if so, how tight it might be) even in mice.

Authors’ response:* We have to disagree about female germ line being repressed in relation to male germ line, as we discussed in the manuscript.*

*We agree about the possible low transcription and replication activity in immature oocytes.*

The authors note later that not all ROS are derived from the mitochondria, but that point seems salient here too. NAD (P)H oxidase may make a far more important contribution. If ROS signals are important for oocyte development, where are they actually coming from? This section implies that they come from mitochondria, but the outer mitochondrial membrane is apparently replete with peroxidise enzymes, and mitochondrial ROS seem to contribute little to cytosolic redox state. It is also hard to be sure that fluorescent probes really reflect ROS leak. For example, the cpYFP probe, claimed to reflect superoxide ‘flashes’, almost certainly reflects pH changes instead (Schwarzlander et al. 2014).

Authors’ response:* We modified the text accordingly.*

A more general point: mitochondrial ROS leakage is typically higher when the mitochondria are in state IV than state III, as fast oxygen consumption lowers the reduction state of the complexes I and III. It’s not correct to assume that usage necessarily leads to more ROS leak, but it might nonetheless be true that usage contributes to a higher mutation rate as a result of greater mtDNA turnover and copying errors.

Authors’ response:* We agree. We integrated this comment in the text.*

Page 15. Mechanisms of preventing Muller’s ratchet and selection for functional mitochondria. I agree that there is some form of selection for functional mitochondria, but that is not necessarily incompatible with template mitochondria, so long as there is UPI and a bottleneck. Both these processes increase the clonality of oocyte mitochondria. That means it is more likely that the mtDNA will be identical in primordial oocytes and the surrounding nurse cells. The function of nurse cells can then be ‘tested’ against the new nuclear background, safe in the knowledge that the oocyte mtDNA is identical. UPI and a bottleneck ensure a tight correlation between mtDNA genotype and phenotype, hence make indirect selection possible. I’m not advocating this viewpoint, just noting that it is possible. There is strong evidence of selection for mitonuclear coadaptation, so I agree that some form of selection must have taken place.

Authors’ response:* We think that the proposed rationale holds only in species in which nurse cells have the same origin as germ cells, like for example in Drosophila. In this organism, the Balbiani body mitochondria seem to reach the oocyte from the nurse cells through the fusome. In species with a somatic derivation of nurse cells (e.g. lizards) this viewpoint is more problematic.*

I would say that the evidence for selective elimination of mtDNA mutations over generations is quite weak. Certainly, the frequency of severely detrimental mtDNA mutations does fall over a number of generations, but these data are as striking for the small degree of bias as for the fact that it happens. The same studies show that less severe mutations are barely eliminated by selection at all. There are also problems with other forms of selection. Autophagy and mitophagy may well be selective for damaged mitochondria, but there is very little consensus in the literature about whether this is really true, and almost zero consensus about mechanism. PINK-1 and Parkin only function if membrane potential is lost altogether, which is an extreme endpoint not seen in this study. I personally doubt that selection for replication speed would tend to favour wild-type mitochondria. There is a very substantial literature in yeast and many other organisms including mice and humans showing that respiratory deficient mutant mitochondria have a proliferative advantage of wild type. A recent example in mice is Burgstaller et al. 2014). Again, it is not beyond the bounds of possibility that selection for replication speed favours wild type, but at the moment there is precious little evidence supporting that, and much against.

Authors’ response:* Even if we are more positive than Prof. Lane about the evidence supporting the elimination of mtDNA mutations over generations, we agree that it is a difficult issue to be addressed.*

My view (simply my attempt to make head or tail of all this, not a prescription for changes) is that UPI, random segregation and bottlenecks all increase the variance between oocytes, and restrict the variance between mitochondria within the same oocyte (i.e. increase clonality). That facilitates selection at the level of cells – cells with good function survive, cells with poor function die by apoptosis.

Authors’ response:* We agree with this.*

Selection could be direct (on oocytes) or indirect (on nurse cells), but direct selection will be favoured when the mtDNA mutation rate is low (as may be the case here) and indirect selection will be favoured when the mutation rate is high, possibly leading to the transcriptional suppression of mitochondria in motile, active bilaterians. The fact that there are so many distinct mechanisms of UPI suggests to me that the selection pressure fluctuates, and that paternal leakage or BPI is more common than is claimed in the literature. While this interpretation might not be true, I don’t think this paper forces me to change it yet.

Authors’ response:* As we reported above, bivalves show a very high DNA polymorphism, so a low mutation rate is unlikely. That said, this represent an interesting view, and a testable hypothesis.*

## Reviewer #2: professor Fedor S Severin

The manuscript presents a set of interesting observations, but it also contains many logical gaps and is very difficult to read. Major points:

In the abstract the authors say that their aim is to test the hypothesis of “division of labor” in species with maternally inherited mtDNA. In fact, they work with DUI animal and essentially show the absence of the “division of labor”.

Authors’ response:* We thank Prof. Severin for the useful comments on the manuscript. We tried to make the narrative clearer and to fill the logical gaps. The hypothesis we wanted to test was the repression of OXPHOS in germ line mitochondria. We rephrased the text to clarify this point.*

In the conclusions part of the abstract the authors claim that they showed that “2) both mitochondrial OXPHOS activity and ROS production are required for oocyte and 3 embryo development”. While the role of OXPHOS in oocyte development is obvious, the arguments for possible function of ROS in this process are not clear. A burst of ROS during oocyte development (refs 60–63 of the manuscript) could be a side effect as there are no proofs presented for functional role of it.

Authors’ response:* We agree. We modified the text accordingly.*

In the conclusions part of the abstract it also says that “3) ROS may not be the main cause of mtDNA mutation…” Apparently, this is not a conclusion, this is rather a hypothesis which is neither supported nor addressed by their experiments.

Authors’ response:* Yes, it was just a hypothesis proposed as a conclusion of the discussion about our results and the results in the literature. We rephrased accordingly.*

I suggest, as an option, the following way to put the data into a story.

First, present some evidence from the literature showing that in species with maternally inherited mtDNA mitochondria in oocyte are less active than in the sperm. Next, motivate their experiments: as in DUI animals, it seems that it does not make sense to suppress oocyte mitochondria (the damaged ones will come with the sperm anyway), the hypothesis the authors are testing is that mitochondrial activity is approximately the same in male and female gametes. Their data support this hypothesis, leading to the next question: How DUI animals cope with the OXPHOS-induced mitochondrial damage in the gametes? One could expect that the levels of the expression of antioxidant enzymes in the gametes of DUI animals are higher than in the gametes of the ones with maternally inherited mtDNA. The concluding part of the revised could address this issue, for example, comparing the transcriptome data obtained by the authors in their experimental system with the published ones for species with maternally inherited mtDNA.

Authors’ response:* The manuscript was organised in the actual order in which things happened. Following the results published recently for other animals, we wanted to test the ‘division of labour’ hypothesis in our system. We expected other results (more in line with a functional silencing of germ line mitochondria), but that did not happen. So we decided to review the existing literature to collect supporting/contrasting evidence and to formulate an alternative hypothesis. For these reasons and for a better understanding of the methodology choices we made, we prefer to maintain the present order. Nevertheless, we liked the summary proposed above by Prof. Severin, so we integrated it in the Conclusions section.*

*The suggested transcriptomic analysis is actually very interesting and we have it already scheduled.*

The authors also present some evidence that the mitochondrial function changes significantly during the development of the gametes. They seem to imply that these variations improve the selection of functional mitochondria during the maturation. To me, this obscures the main story and should be developed into a separate manuscript.

Authors’ response:* We think that this is a central point, and that, in order to obtain comparable data, it is very important to be clear about the analyzed developmental stage. See also the discussion with Prof. Lane above.*

Minor points:

The statement « deltaPSIm appears to be a good proxy for OXPHOS activity» used by authors is not completely true: mitochondria with inhibited ATP-synthase could show high deltaPSI and zero OXPHOS activity.

Authors’ response:* We clarified this point in the manuscript.*

Authors regard metabolically active mitochondria as a potential site of ROS production. However, it is not necessarily so: normally functioning mitochondria can be a site of ROS quenching (see Starkov 2008, doi:10.1196/annals.1427.015).

Authors’ response:* We agree and we added this statement and the related reference in the manuscript.*

## Reviewer #3: professor Fyodor Kondrashov

I am a big fan of the study system selected by the authors: the bi-parental mitochondrial transmission in mollusks. The recent evolution of a male-inherited mitochondria, and an apparent lack of a cross talk of the male and female lines, provides a wonderful model for studying the evolution and function of mitochondria.

Authors’ response:* We thank Prof. Kondrashov for the review. Obviously we totally agree about DUI being a wonderful model. However, we would like to underline that the evolution of male-inherited mitochondria is quite ancient (see answers to Prof. Lane’s comments).*

The authors study one of the crucial aspects of the male-transmitted mitochondria, to which degree the male mitochondria differs in expression from its female counterpart in different gametes, finding some differences. While I cannot attest to the experiments, it appears that it is one of the important questions that can be addressed in this system.

While the authors provide a good general overview of the issues involved (ROS, Muller’s ratchet, etc.), I think that the Conclusion section misses a chance to succinctly summarize their main results.

Authors’ response:* We rewrote the Conclusions section accordingly.*

Also, given that the authors discuss issues of selection in the mitochondrial lineages, I was disappointed to see that there was no sequence analysis performed on the relatively large number of sequences of both male and female mitochondrial available in GenBank. The authors could have tested directly the strength of selection (measured by dn/ds, for example) in male versus female, versus exclusively female mitochondrial lineages.

Authors’ response:* This is actually a very interesting point, but an appropriate discussion would require a dedicated paper. Recently, all the knowledge available about rates of evolution and selection in DUI organisms has been thoroughly reviewed by Zouros (2013). Unfortunately, despite the large number of mtDNA sequences available in GenBank, there is a bias both in the sequenced genes (mostly small fragments of* cytb *or* cox1*) and in species. Another big issue is the absence of polymorphism data in most species (that is, most species have just a single mt genome available). This means that we could easily collect a proper dataset to calculate divergence between species, but then we could not do the same for polymorphism within species. We are now in the process of sequencing multiple individuals belonging to several different DUI and non-DUI bivalve species, hoping to provide new insight into this fundamental topic.*

*References:*

*Zouros E: Biparental Inheritance Through Uniparental Transmission: The Doubly Uniparental Inheritance (DUI) of Mitochondrial DNA. Evol Biol 2013, 40: 1–31.*

Minor points:

“Of course, no implication for male-transmitted mtDNA viability was ever taken into consideration” – Change to “was never”

Authors’ response:* We rephrased the sentence.*

## Abbreviations

mtDNA: mitochondrial DNA
OXPHOS: Oxidative phosphorylation
ROS: Reactive oxygen species
DUI: Doubly uniparental inheritance of mitochondria
M-type: Sperm-transmitted mtDNA/mitochondrion
F-type: Egg-transmitted mtDNA/mitochondrion
SMI: Strictly maternal inheritance of mitochondria
ATP: Adenosine triphosphate
TEM: Transmission electron microscope
nm: Nanometers (1 × 10^−9^ m)
qPCR: Quantitative polymerase chain reaction
*18S*: 18S ribosomal RNA
*cytb*: Cytochrome b
Δψm: Mitochondrial membrane potential
μm: Micrometers (1 × 10^−6^ m)
Bb: Balbiani body (“mitochondrial cloud”)
PGCs: Primordial germ cells

## References

[1] Timmis JN, Ayliffe MA, Huang CY, Martin W (2004). Endosymbiotic gene transfer: organelle genomes forge eukaryotic chromosomes. Nat Rev Genet.

[2] Breton S, Milani L, Ghiselli F, Guerra D, Stewart DT, Passamonti M. A resourceful genome: updating the functional repertoire and evolutionary role of animal mitochondrial DNAs. Trends Genet. 2014;30:555-64. 10.1016/j.tig.2014.09.002 25263762

[3] Allen JF (1993). Control of gene expression by redox potential and the requirement for chloroplast and mitochondrial genomes. J Theor Biol.

[4] Allen JF (2003). The function of genomes in bioenergetic organelles. Phil Trans R Soc Lond B.

[5] Lane N, Martin W (2010). The energetics of genome complexity. Nature.

[6] Lane N (2011). Energetics and genetics across the prokaryote-eukaryote divide. Biol Direct.

[7] Rand DM, Haney RA, Fry AJ (2004). Cytonuclear coevolution: the genomics of cooperation. Trends Ecol Evol.

[8] Burton RS, Barreto FS (2012). A disproportionate role for mtDNA in Dobzhansky-Muller incompatibilities?. Mol Ecol.

[9] Wolff JN, Ladoukakis ED, Enríquez JA, Dowling DK (2014). Mitonuclear interactions: evolutionary consequences over multiple biological scales. Phil Trans R Soc B.

[10] Hoekstra RF, Togashi T, Cox PA (2011). Nucleo-cytoplasmic conflict and the evolution of gamete dimorphism. The Evoltion of Anisogamy. A fundamental phenomenon underlying sexual selection.

[11] Andreyev AY, Kushnareva YE, Starkov AA (2005). Mitochondrial metabolism of reactive oxygen species. Biochemistry (Mosc).

[12] Harman D (1956). Aging: a theory based on free radical and radiation chemistry. J Gerontol.

[13] Beckman KB, Ames BN (1998). The free radical theory of aging matures. Physiol Rev.

[14] Starkov AA (2008). The role of mitochondria in reactive oxygen species metabolism and signaling. Ann N Y Acad Sci.

[15] Lane N (2011). Mitonuclear match: optimizing fitness and fertility over generations drives ageing within generations. Bioessays.

[16] Cosmides LM, Tooby J (1981). Cytoplasmic inheritance and intragenomic conflict. J Theor Biol.

[17] Hurst LD (1992). Intragenomic conflict as an evolutionary force. Proc Royal Soc Lond B.

[18] Allen JF (1996). Separate sexes and the mitochondrial theory of ageing. J Theor Biol.

[19] Allen JF, de Paula WMB (2013). Mitochondrial genome function and maternal inheritance. Biochem Soc T.

[20] de Paula WBM, Lucas CH, Agip A-N A, Vizcay-Barrena G, Allen JF (2013). Energy, ageing, fidelity and sex: oocyte mitochondrial DNA as a protected genetic template. Phil Trans R Soc B.

[21] de Paula WBM, Agip A-N A, Missirlis F, Ashworth R, Vizcay-Barrena G, Lucas CH (2013). Female and male gamete mitochondria Are distinct and complementary in transcription, structure, and genome function. Genome Biol Evol.

[22] Zouros E (2013). Biparental inheritance through uniparental transmission: the doubly uniparental inheritance (DUI) of mitochondrial DNA. Evol Biol.

[23] Saavedra C, Reyero MI, Zouros E (1997). Male-dependent doubly uniparental inheritance of mitochondrial DNA and female-dependent sex-ratio in the mussel *Mytilus galloprovincialis*. Genetics.

[24] Sutherland B, Stewart DT, Kenchington E, Zouros E (1998). The fate of paternal mitochondrial DNA in developing female mussels, *Mytilus edulis*: implications for the mechanism of doubly uniparental inheritance of mitochondrial DNA. Genetics.

[25] Milani L, Ghiselli F, Maurizii MG, Passamonti M (2011). Doubly uniparental inheritance of mitochondria as a model system for studying germ line formation. PLoS One.

[26] Milani L, Ghiselli F, Passamonti M (2012). Sex-linked mitochondrial behavior during early embryo development in *Ruditapes philippinarum* (Bivalvia Veneridae) a species with the Doubly Uniparental Inheritance (DUI) of mitochondria. J Exp Zool Part B.

[27] Breton S, Ghiselli F, Passamonti M, Milani L, Stewart DT, Hoeh WR (2011). Evidence for a fourteenth mtDNA-encoded protein in the female-transmitted mtDNA of marine mussels (Bivalvia: Mytilidae). PLoS One.

[28] Venetis C, Theologidis I, Zouros E, Rodakis GC (2006). No evidence for presence of maternal mitochondrial DNA in the sperm of *Mytilus galloprovincialis* males. Proc Biol Sci.

[29] Ghiselli F, Milani L, Passamonti M (2011). Strict sex-specific mtDNA segregation in the germline of the DUI species *Venerupis philippinarum* (Bivalvia Veneridae). Mol Biol Evol.

[30] Passamonti M, Ghiselli F (2009). Doubly uniparental inheritance: two mitochondrial genomes, on precious model for organelle DNA inheritance and evolution. DNA Cell Biol.

[31] Milani L, Ghiselli F, Nuzhdin SV, Passamonti M (2013). Nuclear Genes with Sex Bias in *Ruditapes philippinarum* (Bivalvia, Veneridae): Mitochondrial Inheritance and Sex Determination in DUI Species. J Exp Zool Part B.

[32] Larkman AU (1984). The fine structure of mitochondria and the mitochondrial cloud during oogenesis on the sea anemone *Actinia*. Tissue Cell.

[33] Hertig AT, Adams EC (1967). Studies on the human oocyte and its follicle. I. Ultrastructural and histochemical observations on the primordial follicle stage. J Cell Biol.

[34] Motta PM, Nottola SA, Makabe S, Heyn R (2000). Mitochondrial morphology in human fetal and adult female germ cells. Hum Reprod.

[35] Van Blerkom J, Davis P, Lee J (1995). ATP content of human oocytes and developmental potential and outcome after in-vitro fertilization and embryo transfer. Hum Reprod.

[36] Dumollard R, Duchen M, Carroll J (2007). The role of mitochondrial function in the oocyte and embryo. Curr Top Dev Biol.

[37] Ghiselli F, Milani L, Guerra D, Chang PL, Breton S, Nuzhdin SV (2013). Structure, transcription and variability of metazoan mitochondrial genome. Perspectives from an unusual mitochondrial inheritance system. Genome Biol Evol.

[38] Fabioux C, Huvet A, Lelong C, Robert R, Pouvreau S, Daniel JY (2004). Oyster vasa-like gene as a marker of the germline cell development in *Crassostrea gigas*. Biochem Bioph Res Co.

[39] Faccenda D, Tan CH, Duchen MR, Campanella M (2013). Mitochondrial IF1 preserves cristae structure to limit apoptotic cell death signaling. Cell Cycle.

[40] Wilding M, Carotenuto R, Infante V, Dale B, Marino M, Di Matteo L (2001). Confocal microscopy analysis of the activity of mitochondria contained within the ’mitochondrial cloud’ during oogenesis in *Xenopus laevis*. Zygote.

[41] Van Blerkom J, Davis P (2007). Mitochondrial signaling and fertilization. Mol Hum Reprod.

[42] Zhang Y-Z, Ouyang Y-C, Hou Y, Schatten H, Chen D-Y, Sun Q-Y (2008). Mitochondrial behavior during oogenesis in zebrafish: a confocal microscopy analysis. Develop Growth Differ.

[43] Lee S-K, Zhao M-H, Kwon J-W, Li Y-H, Lin Z-L, Jin Y-X (2014). The association of mitochondrial potential and copy number with Pig oocyte maturation and developmental potential. J Reprod Dev.

[44] Noguchi T, Koizumi M, Hayashi S (2011). Sustained elongation of sperm tail promoted by local remodeling of giant mitochondria in *drosophila*. Curr Biol.

[45] Nishimura Y, Yoshinari T, Naruse K, Yamada T, Sumi K, Mitani H (2006). Active digestion of sperm mitochondrial DNA in single living sperm revealed by optical tweezers. Proc Natl Acad Sci U S A.

[46] Guan J, Kinoshita M, Yuan L (2009). Spatiotemporal association of DNAJB13 with the annulus during mouse sperm flagellum development. BMC Dev Biol.

[47] Celeghini ECC, Nascimento J, Raphael CF, Andrade AFC, Arruda RP (2010). Simultaneous assessment of plasmatic, acrosomal, and mitochondrial membranes in ram sperm by fluorescent probes. Arq Bras Med Vet Zootec.

[48] Müller M, Mentel M, van Hellemond JJ, Henze K, Woehle C, Gould SB (2012). Biochemistry and evolution of anaerobic energy metabolism in eukaryotes. Microbiol Mol Biol Rev.

[49] Mann T, Rothschild (1950). Carbohydrate and Adenosinetriphosphate in Sea-urchin semen. Nature.

[50] Mita M, Nakamura M (1998). Energy metabolism of sea urchin spermatozoa: an approach based on echinoid phylogeny. Zoolog Sci.

[51] Ramalho-Santos J, Varum S, Amaral S, Mota PC, Sousa AP, Amaral A (2009). Mitochondrial functionality in reproduction: from gonads and gametes to embryos and embryonic stem cells. Hum Reprod Update.

[52] Van Blerkom J (2011). Mitochondrial function in the human oocyte and embryo and their role in developmental competence. Mitochondrion.

[53] Ge H, Tollner TL, Hu Z, Dai M, Li X, Guan H (2012). The importance of mitochondrial metabolic activity and mitochondrial DNA replication during oocyte maturation in vitro on oocyte quality and subsequent embryo developmental competence. Mol Reprod Dev.

[54] Wanet A, Arnould T, Renard P, Lou PH, Peterson N, Lou P-H, Peterson N (2012). Mitochondrial involvement in Stemness and stem cell differentiation. Cellular bioenergetics in health and disease: New perspective in mitochondrial biology.

[55] Nichols DG, Ferguson SJ (2002). Bioenergetics.

[56] Zorov DB, Isaev NK, Plotnikov EY, Zorova LD, Stelmashook EV, Vasileva AK (2007). The mitochondrion as Janus Bifrons. Biochem Moscow.

[57] Wilding M, Dale B, Marino M, di Matteo L, Alviggi C, Pisaturo ML (2001). Mitochondrial aggregation patterns and activity in human oocytes and preimplantation embryos. Hum Reprod.

[58] Wang X, Sharma RK, Gupta A, George V, Thomas AJ, Falcone T (2003). Alterations in mitochondria membrane potential and oxidative stress in infertile men: a prospective observational study. Fertil Steril.

[59] Tsukagoshi H, Busch W, Benfey PN (2010). Transcriptional regulation of ROS controls transition from proliferation to differentiation in the root. Cell.

[60] Cottet-Rousselle C, Ronot X, Leverve X, Mayol J-F (2011). Cytometric assessment of mitochondria using fluorescent probes. Cytom Part A.

[61] Le Belle JE, Orozco NM, Paucar AA, Saxe JP, Mottahedeh J, Pyle AD (2011). Proliferative neural stem cells have high endogenous ROS levels that regulate self-renewal and neurogenesis in a PI3K/Akt-dependant manner. Cell Stem Cell.

[62] Hennet ML, Combelles CM (2012). The antral follicle: a microenvironment for oocyte differentiation. Int J Dev Biol.

[63] Martín M, Distefano A, Zabaleta E, Pagnussat G (2013). New insights into the functional roles of reactive oxygen species during embryo sac development and fertilization in *Arabidopsis thaliana*. Plant Signal Behav.

[64] Barja G (2013). Updating the mitochondrial free radical theory of aging: an integrated view, Key aspects, and confounding concepts. Antioxid Redox Sign.

[65] Viña J, Borras C, Abdelaziz KM, Garcia-Valles R, Gomez-Cabrera MC (2013). The free radical theory of aging revisited: the cell signaling disruption theory of aging. Antioxid Redox Signal.

[66] Russo R, Monaco D, Rubessa M, El-Bahrawy KA, El-Sayed A, Martino NA (2014). Confocal fluorescence assessment of bioenergy/redox status of dromedary camel (*Camelus dromedarius*) oocytes before and after in vitro maturation. Reprod Biol Endocrin.

[67] Galtier N, Jobson RW, Nabholz B, Glémin S, Blier PU (2009). Mitochondrial whims: metabolic rate, longevity and the rate of molecular evolution. Biol Lett.

[68] Speakman JR, Garratt M (2013). Oxidative stress as a cost of reproduction: beyond the simplistic trade-off model. Bioessays.

[69] Trifunovic A, Wredenberg A, Falkenberg M, Spelbrink JN, Rovio AT, Bruder CE (2004). Premature ageing in mice expressing defective mitochondrial DNA polymerase. Nature.

[70] Kujoth GC, Hiona A, Pugh TD, Someya S, Panzer K, Wohlgemuth SE (2005). Mitochondrial DNA mutations, oxidative stress, and apoptosis in mammalian aging. Science.

[71] Nabholz B, Glémin S, Galtier N (2008). Strong variations of mitochondrial mutation rate across mammals-the longevity hypothesis. Mol Biol Evol.

[72] Kennedy SR, Salk JJ, Schmitt MW, Loeb LA (2013). Ultra-sensitive sequencing reveals an age-related increase in somatic mitochondrial mutations that are inconsistent with oxidative damage. PLoS Genet.

[73] Itsara LS, Kennedy SR, Fox EJ, Yu S, Hewitt JJ, Sanchez-Contreras M (2014). Oxidative stress is not a major contributor to somatic mitochondrial DNA mutations. PLoS Genet.

[74] Theiss AL, Idell RD, Srinivasan S, Klapproth JM, Jones DP, Merlin D (2007). Prohibitin protects against oxidative stress in intestinal epithelial cells. FASEB J.

[75] Kienhöfer J, Häussler DJ, Ruckelshausen F, Muessig E, Weber K, Pimentel D (2009). Association of mitochondrial antioxidant enzymes with mitochondrial DNA as integral nucleoid constituents. FASEB J.

[76] Bogenhagen DF (1819). Mitochondrial DNA nucleoid structure. Biochim Biophys Acta.

[77] Gilkerson R, Bravo L, Garcia I, Gaytan N, Herrera A, Maldonado A (2013). The mitochondrial nucleoid: integrating mitochondrial DNA into cellular homeostasis. Cold Spring Harb Perspect Biol.

[78] Wardman P (2007). Fluorescent and luminescent probes for measurement of oxidative and nitrosative species in cells and tissues: progress, pitfalls, and prospects. Free Radic Biol Med.

[79] Schwarzländer M, Wagner S, Ermakova YG, Belousov VV, Radi R, Beckman JS (2014). The ‘mitoflash’ probe cpYFP does not respond to superoxide. Nature.

[80] Forman HJ (2009). Critical methods in free radical biology & medicine. Free Radical Bio Med.

[81] Kohen R, Nyska A (2002). Oxidation of biological systems: oxidative stress phenomena, antioxidants, redox reactions, and methods for their quantification. Toxicol Pathol.

[82] Shoubridge EA, Wai T (2007). Mitochondrial DNA and the mammalian oocyte. Curr Top Dev Biol.

[83] Mishra P, Chan DC (2014). Mitochondrial dynamics and inheritance during cell division, development and disease. Nat Rev Mol Cell Biol.

[84] Chinnery PF, Thorburn DR, Samuels DC, White SL, Dahl HM, Turnbull DM (2000). The inheritance of mitochondrial DNA heteroplasmy: random drift, selection or both?. Trends Genet.

[85] Fan W, Waymire KG, Narula N, Li P, Rocher C, Coskun PE (2008). A mouse model of mitochondrial disease reveals germline selection against severe mtDNA mutations. Science.

[86] Shoubridge EA, Wai T (2008). Medicine. Sidestepping mutational meltdown. Science.

[87] Freyer C, Cree LM, Mourier A, Stewart JB, Koolmeister C, Milenkovic D (2012). Variation in germline mtDNA heteroplasmy is determined prenatally but modified during subsequent transmission. Nat Genet.

[88] Hill JH, Chen Z, Xu H (2014). Selective propagation of functional mitochondrial DNA during oogenesis restricts the transmission of a deleterious mitochondrial variant. Nat Genet.

[89] Kloc M, Bilinski S, Etkin LD (2004). The Balbiany body and germ cell determinants: 150 years later. Curr Top Dev Biol.

[90] Pepling ME, Wilhelm JE, O’Hara AL, Gephardt GW, Spradling AC (2007). Mouse oocytes within germ cell cysts and primordial follicles contain a Balbiani body. Proc Natl Acad Sci U S A.

[91] Guraya SS (1979). Recent advances in the morphology, cytochemistry and function of Balbiani’s vitelline body in animal oocytes. Int Rev Cytol.

[92] Pepling ME, Spradling AC (1998). Female mouse germ cells form synchronously dividing cysts. Development.

[93] Cox RT, Spradling AC (2003). A Balbiani body and the fusome mediate mitochondrial inheritance during *Drosophila* oogenesis. Development.

[94] Cox RT, Spradling AC (2006). Milton controls the early acquisition of mitochondria by *Drosophila* oocytes. Development.

[95] Zhou RR, Wang B, Wang J, Schatten H, Zhang YZ (2010). Is the mitochondrial cloud the selection machinery for preferentially transmitting wild-type mtDNA between generations? Rewinding Müller’s ratchet efficiently. Curr Genet.

[96] Lim AK, Kai T (2007). Unique germ-line organelle, nuage, functions to repress selfish genetic elements in *Drosophila melanogaster*. Proc Natl Acad Sci U S A.

[97] Wai T, Teoli D, Shoubridge EA (2008). The mitochondrial DNA genetic bottleneck results from replication of a subpopulation of genomes. Nat Genet.

[98] Dujon B, Strathern JN, Jones EW, Broach JR (1981). Mitochondrial genetics and functions. The molecular biology of the yeast saccharomyces.

[99] Yoneda M, Chomyn A, Martinuzzi A, Hurko O, Attardi G (1992). Marked replicative advantage of human mtDNA carrying a point mutation that causes the MELAS encephalomyopathy. Proc Natl Acad Sci U S A.

[100] Krakauer DC, Mira A (1999). Mitochondria and germ-cell death. Nature.

[101] Milani L, Ghiselli F, Iannello M, Passamonti M (2014). Evidence for somatic transcription of male-transmitted mitochondrial genome in the DUI species *Ruditapes philippinarum* (Bivalvia: Veneridae). Curr Genet.

[102] Rozen S, Skaletsky H (2000). Primer3 on the WWW for general users and for biologist programmers. Methods Mol Biol.

[103] Livak KJ, Schmittgen TD (2001). Analysis of relative gene expression data using real-time quantitative PCR and the 2 (−Delta Delta C (T)) Method. Methods.

[104] Pendergrass W, Wolf N, Poot M (2004). Efficacy of MitoTracker GreenTM and CMXRosamine to measure changes in mitochondrial membrane potentials in living cells and tissues. Cytometry Part A.

[105] Casper-Lindley C, Kimura S, Saxton DS, Essaw Y, Simpson I, Tan V (2011). Rapid Fluorescence-Based Screening for *Wolbachia* Endosymbionts in *Drosophila* Germ Line and Somatic Tissues. Appl Environ Microb.

[106] Marlow FL, Mullins MC (2008). Bucky ball functions in Balbiani body assembly and animal–vegetal polarity in the oocyte and follicle cell layer in zebrafish. Dev Biol.

[107] Yu Y, Dumollard R, Rossbach A, Lai FA, Swann K (2010). Redistribution of mitochondria leads to bursts of ATP production during spontaneous mouse oocyte maturation. J Cell Physiol.

[108] Reyes M, Palomino J, Parraguez VH, Hidalgo M, Saffie P (2011). Mitochondrial distribution and meiotic progression in canine oocytes during in vivo and in vitro maturation. Theriogenology.

